# Periocular Aging Across Populations and Esthetic Considerations: A Narrative Review

**DOI:** 10.3390/jcm14020535

**Published:** 2025-01-16

**Authors:** Brendan K. Tao, Fahad R. Butt, Thanansayan Dhivagaran, Michael Balas, Navdeep Nijhawan, Georges Nassrallah, Ahsen Hussain, Edsel B. Ing

**Affiliations:** 1 Faculty of Medicine, The University of British Columbia, Vancouver, BC V6T 1Z3, Canada; brendan.tao2016@gmail.com; 2 Schulich School of Medicine & Dentistry, University of Western Ontario, London, ON N6A 5C1, Canada; fbutt2027@meds.uwo.ca (F.R.B.); tdhivagaran2027@meds.uwo.ca (T.D.); 3 Department of Ophthalmology and Vision Sciences, University of Toronto, Toronto, ON M5T 3A9, Canada; 1michaelbalas@gmail.com (M.B.); navnijhawan@hotmail.com (N.N.); gnassra1@jhmi.edu (G.N.); 4 Department of Ophthalmology, Dalhousie University, Halifax, NS B3H 4R2, Canada; ahsen@dal.ca; 5 Department of Ophthalmology & Visual Sciences, University of Alberta, Edmonton, AB T5H 3V9, Canada

**Keywords:** oculoplastic surgery, oculofacial surgery, aging, esthetics, minimally invasive procedures, diversity equity inclusion

## Abstract

As the face ages, the skin, fat, muscle, and fascia descend, and the underlying bone, cartilage, and teeth may lose mass. Oculofacial aging is a multifactorial process that is influenced by genetic, environmental, and lifestyle factors. This review summarizes the patterns of oculofacial aging that are observed across populations, including variations in periorbital hollowing, eyelid ptosis, and skin elasticity. Evidence indicates significant variability in aging patterns between sex- and race-based subgroups. Nonetheless, there remains a paucity of research on the progression of aging in some under-studied demographic groups. Signs of oculofacial aging often become apparent to patients well before these changes reach full maturity in later years, leading many to seek early esthetic interventions. Others may present with more advanced signs of aging, motivating a diverse range of therapeutic options. We discuss minimally invasive esthetic interventions to mitigate the signs of aging, which may include botulinum toxin injections, dermal fillers, applied energy-based treatments (e.g., lasers), and emerging techniques such as micro-focused ultrasound and platelet-rich plasma therapies. We review evidence on outcomes related to patient satisfaction and quality of life following esthetic interventions for oculofacial aging. Finally, we outline ethical considerations and challenges faced with the delivery of esthetic surgery, including treatment complications and the influence of social media. This review provides a comprehensive overview of oculofacial aging patterns, its management, and important considerations for the provision of esthetic oculofacial treatment.

## 1. Terminology and Scope

Oculofacial aging refers to the morphological evolution of orbital and periocular structures in response to senescence-related changes, for which the underlying etiology is multifactorial and may include genetic and environmental factors [1,2]. Although the advanced stages of oculofacial aging are well recognized, the signs of this progressive phenomenon may begin to manifest in early adulthood. The signs of oculofacial aging may involve periorbital hollowing, eyelid ptosis, and reduced skin elasticity [2].

In this narrative review, a manual review of PubMed (search terms: “oculofacial ageing”, “peri-orbital ageing”, “facial ageing”) and citation lists of considered studies was conducted to summarize the patterns of oculofacial aging across populations and provide an overview of minimally invasive esthetic interventions for the treatment of age-related periocular changes. Finally, we review the literature on post-intervention outcomes related to patient satisfaction and quality of life, followed by a discussion on ethical considerations and challenges associated with the provision of periocular esthetic treatment.

## 2. Patterns of Oculofacial Aging

### 2.1. Pathophysiology

Genetic factors, which play a pivotal role in determining the trajectory of oculofacial aging, influence the production of collagen and elastin proteins essential for maintaining skin elasticity and firmness [3]. Several genes underline the structure and function of collagen and elastin, where different polymorphisms of these genes between individuals have been suggested to confer varying resistance to aging [4]. In extreme cases, conditions of anomalous or deficient matrix proteins may predispose individuals to accelerated skin aging patterns [3].

To date, human collagen is thought to be influenced by a family of 46 genes [4]. Skin is primarily composed of fibrillar collagen, which is predominantly composed of type I collagen (contributed by the COL1A1 and COL1A2 genes), with smaller contributions from type III (involving the COL3A1 gene) and type V (involving COL5A1 and COL5A2) collagen [5]. In contrast, elastin, encoded by the ELN gene, includes 34 exons, 6 of which (22, 23, 24, 26A, 32, and 33) are alternatively spliced to modulate gene expression [6]. Notably, exposure to ultraviolet (UV) light and high temperatures has been associated with the increased expression of exon 26A—normally excluded—leading to structural changes in elastin fibers linked to photoaged skin [7].

Besides determining matrix protein integrity, genetic factors also predict general resistance to inflammatory and oxidative stressors [8]. The features of age-related changes, including cellular and immune senescence, are driven by chronic systemic inflammation [9]. For instance, higher levels of superoxide dismutase 2 (SOD2), an enzyme responsible for neutralizing reactive oxygen species, may enhance resistance to oxidative stressors, potentially lowering the risk of age-related outcomes [10]. Moreover, genome-wide association studies suggest a median heritability of 37% across a wide array of immune traits and cell types, which appear to be key determinants in the inflammatory aging process [11].

Genetic factors further influence the content and type of cutaneous melanin, a protective factor against UV radiation [12]. Clinically, the Fitzpatrick skin type stratifies the range of cutaneous melanin content from types I to VI [13]. Higher Fitzpatrick classifications (IV to VI) demonstrate delayed photoaging effects due to elevated UV protection conferred by higher melanin contents [12,14].

Besides genetic factors, environmental risk factors also play a significant role in oculofacial aging. Sun exposure is among the most prominent risk factors for skin aging. Increased exposure to sun-derived UV light accelerates photoaging, manifesting as cutaneous wrinkling, altered skin texture, and hyperpigmentation [15]. UV-B radiation damages the epidermis, causing DNA damage, pigmentation changes, and carcinogenesis, while UV-A wavelengths penetrate deeper into the dermis, leading to collagen breakdown and proteinase production [16]. These processes involve the generation of reactive oxygen species, thereby increasing oxidative stress and resulting in apoptosis, melanogenesis, and damage to DNA and proteins (e.g., collagen) in cutaneous soft tissue [17]. Further, exposure to air pollutants (e.g., nitrogen dioxide, ozone, and polycyclic aromatic hydrocarbons, among other particulates) is another risk factor for skin aging, as it also generates reactive oxygen species, leading to cutaneous inflammation [17]. Various climate factors (e.g., aridity, extreme temperatures, and excess wind or air conditioning exposure) promote trans-epidermal water loss and moisture depletion from the stratum corneum, resulting in dermal irritation and cutaneous wrinkling [18,19].

**Figure 1:**
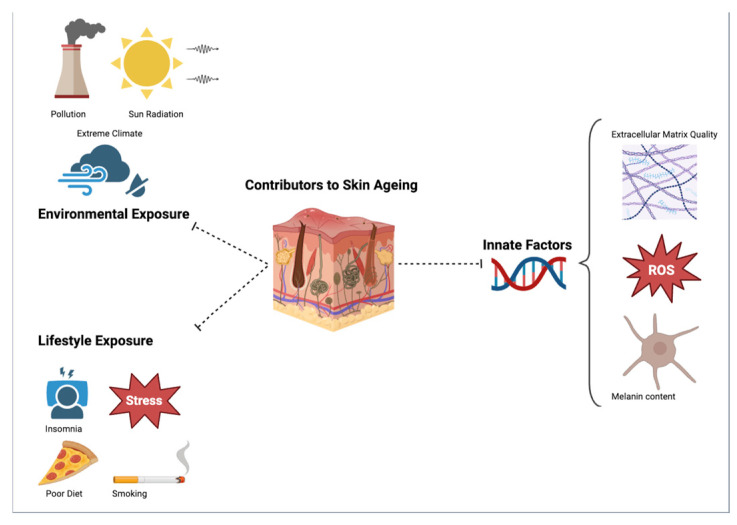
Visual summary of common pathophysiological contributors to periorbital aging. Among these, sun exposure is a leading contributing factor. In this diagram, stress refers to both psychological stress and mechanical stressors to the skin (e.g., facial rubbing, stretching from longstanding edema, repetitive facial expressions, and sleeping prone). Abbreviation: ROS (reactive oxygen species).

Lifestyle exposures compose a third major category of aging risk factors. Of these, heavy smoking has been linked to accelerated periorbital wrinkling, tear-trough hollowing, and under-eye puffiness [20,21]. One mechanism for this association may involve the induced vasoconstriction of the periocular vascular supply [22], which may contribute to a dull ashen-like skin quality with premature periocular wrinkling. Further, smoking has been associated with impaired collagen production and increased synthesis of degrading matrix metalloproteinases, resulting in wrinkling and premature skin aging [23,24]. Comparatively, less consistent evidence supports alcohol ingestion as a risk factor for facial wrinkling and midface volume loss [25]. Healthy diets emphasizing sufficient fluid, antioxidants (e.g., vitamins A, C, and E), and carotenoid intake are associated with decreased facial wrinkling [26]. Other potential risk factors include chronic stress [27], the stasis of dynamic wrinkling induced by repetitive facial expressions (e.g., squinting, frowning) or distortions during sleep [28], and generally poor sleep hygiene [29]. A visual illustration of the pathophysiological contributors to periorbital aging are depicted in Figure 1, whereas Table 1 summarizes skin-related changes caused by these contributors.

### 2.2. Periorbital Aging Patterns

**Figure 2:**
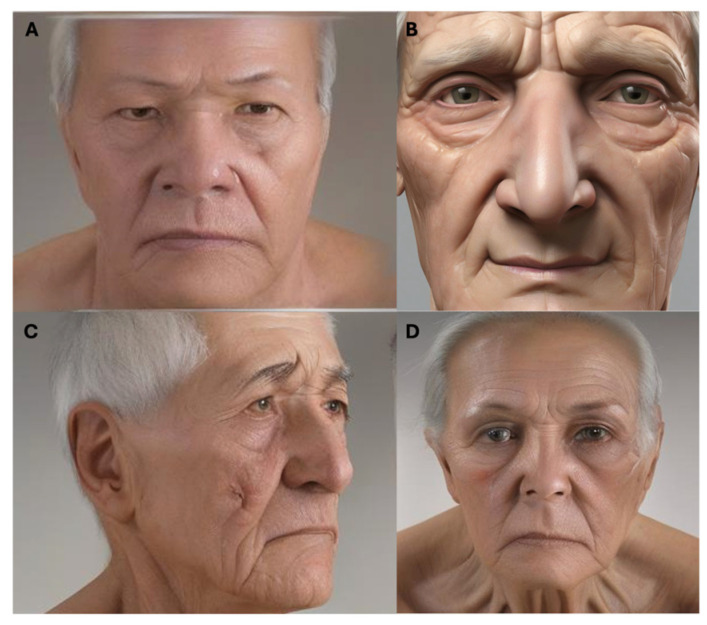
Common clinical examination findings in peri-ocular aging. Images were generated using artificial intelligence to preserve patient confidentiality. (**A**) Brow ptosis, more prominent on the patient’s right side. (**B**) Superior sulcus hollowing with glabellar furrows and lower dermatochalasis with double convexity deformity. (**C**) Prominent right nasojugal folds. (**D**) Suggestion of mild lower lid ectropion with loss of right lateral lid-globe apposition.

Oculofacial aging induces changes in orbital and facial regions throughout ones’s lifespan. Generally, youthful appearances are associated with seamless transitions between facial contours and uniform skin textures, while periocular aging alters the qualities of bony and soft tissue landmarks, resulting in a less cohesive facial structure [2]. The changes in the face with aging can be categorized into those affecting the upper and middle face. Jowls occur from the sagging of the platysma muscle behind the mandibular ligament, but we will not discuss this in detail. Figure 2 depicts common clinical examination findings in peri-ocular aging.

#### 2.2.1. Upper Face: Forehead, Brows, and Upper Eyelids

In the early stages of oculofacial aging, the most notable changes can be seen as decreased skin elasticity and changes in pigmentation, the onset of periorbital hollowing, and mid-eyelid changes. The decreased elasticity of the skin results from a reduction in the number of fibroblasts that synthesize collagen and elastin, thereby increasing the propensity for skin laxity and periocular fine wrinkle lines [1]. There is also periorbital tissue hollowing, particularly between the eyes and the upper medial cheeks, primarily driven by the volume depletion of soft tissue and bone resorption [30,31]. The upper one-third of the face includes the forehead, which has an important role in providing structural support to the eyebrows and eyelids. It is bounded by the hairline (superiorly), the temporal ridge (laterally), and the supraorbital ridge (inferiorly). The frontal and parietal bones contribute to the skeletal structure of the forehead, which undergoes changes over time that contribute to oculofacial aging. The frontal bone enlarges and undergoes curvature distortion at the orbital rim, contributing to an overall flattening of the forehead [32,33,34]. This flattening contributes to the inward projection of the eyebrows and eyelids.

Furthermore, with age, the forehead, temporal, and brow regions undergo lipoatrophy, making the underlying bony structures, such as the supraorbital rims, more pronounced. This loss of subcutaneous fat gives the face a gaunt appearance. Volume loss in the forehead and temple regions also decreases structural support for the lateral brow, resulting in brow ptosis near or below the supraorbital rim [35,36]. Moreover, with subcutaneous fat loss and decreased skin elasticity with aging, the forehead expression muscles become more pronounced, leading to the formation of deep rhytids [37]. Transverse rhytids are formed by the frontalis muscles, which elevate the brow and forehead. The procerus muscle contributes to horizontal glabellar furrows. Vertical rhytids are formed primarily by the corrugator supercillii and depressor supercilii muscles, which pull the brow medially and inferiorly [2].

The eyebrows, eyes, and periorbital areas are a primary site of facial aging and are among the most expressive regions of the face. A youthful appearance in the periocular region is associated with eyebrow positioning above the supraorbital ridge, with forward projection due to underlying retro-orbicularis oculi fat. As stated, lipoatrophy with age causes the brow to fall, due to reduced structural support from decreased fat content and increased skin laxity [38]. Temporal hooding may occur because the medial brow typically descends less than the lateral brow. Next, the upper eyelid undergoes several changes with aging, particularly dermatochalasis, blepharoptosis, and the formation of crow’s feet. The attenuation of the orbital septum causes forward prolapse of the periocular fat in the upper and lower lid. Dermatochalasis is characterized by increased skin laxity and redundancy of the eyelid skin, a hallmark sign of periocular aging [39]. In comparison, blepharoptosis is defined as the descent of the upper eyelid margin, caused by weakening of the levator muscle and its attachments [40]. Periocular ligaments (e.g., orbito-malar ligaments) likewise undergo aging changes, giving rise to deep periorbital hollows that may be striking to patients. Patients may also experience facial volume loss secondary to fat atrophy in the upper and lower eyelids, contrasting with fat prolapse, which may occur independently or in other patients. Lastly, crow’s feet are rhytids radiating from the lateral canthal area, caused by the repeated contraction of the orbicularis oculi muscle over time [41].

#### 2.2.2. Midface: Lower Eyelids and Nose

The midface is the region inferior to the lower eyelids and superior to the oral commissure. Faces that appear youthful have an ogee curve, an S-shaped contour noticeable on the cheek when the face is viewed from an oblique angle [42]. This curve involves a double curve with a single upper convexity formed by the suborbicularis oculi fat and malar fat pad, which converge at the nasolabial fold [2]. With periocular aging, there is gradual volume loss and sagging/descent of the midface, leading to a flattened double convexity and disruption of the naturally occurring ogee curve [2]. The inferior eyelid skin, eyelid fat pads, and both the pretarsal and preseptal portions of the orbicularis oculi muscle shape the first upper convexity. Due to the enlargement and pseudoherniation of orbital fat, the lower eyelid fat pads gradually appear more pronounced, emphasizing the tear trough [2]. In contrast, the suborbicularis oculi fat, malar fat pad, and orbital portion of the orbicularis oculi muscle contribute to the lower convexity. Deeper tissues begin to sag separately from the superficial skin, as areas with underlying facial ligaments, such as the lid–cheek junction, experience reduced gravitational effects. The superficial aspect of the inferior eyelid is highly prone to sun exposure and associated photoaging effects, which may lead to skin malignancies, pigmentation changes, and rhytid formation [2].

Furthermore, the development of age-related eyelid malposition is closely linked to the age-related changes seen in the midface. The laxity or disinsertion observed within the medial or lateral canthal tendons may result in entropion or ectropion due to the inversion or eversion of the inferior eyelid, respectively [2]. Within the midface, sagging can worsen ectropion and lead to various symptoms, including eczematous skin changes, tearing, and foreign body sensation [2].

Regarding the nose, it may appear larger due to alterations in projection, while nearby structures recede as a result of age-related changes [37]. Simultaneous maxillary and nasal bone loss enlarges the piriform aperture, reducing nasal support and causing the columella and lateral cartilage to shift backward, leading to nasal tip ptosis and altering nose projection [2].

#### 2.2.3. Demographic Differences

Males and females exhibit some differences in the oculofacial aging patterns. In terms of aging onset, women experience minimal change by age 30, whereas men experience a significant increase in facial size [43]. Often, after the age of 50, women tend to experience a steeper trajectory of facial aging compared to men, particularly during early menopause [44]. Women experience more rapid soft tissue atrophy compared to men during pre-menopause; however, men exhibit more pronounced volume loss in the periocular area, with more severe lower eyelid sagging [45]. Men also have a reduced innate antioxidant capacity, making them more susceptible to UV-induced cellular damage [45].

**Figure 3:**
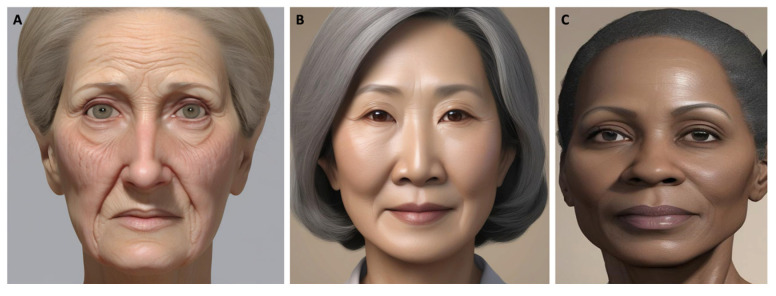
Example of varying female oculofacial aging patterns between population groups. Images were generated using artificial intelligence to preserve patient confidentiality. (**A**) Caucasian. (**B**) East Asian. (**C**) Black or African American. The features of oculofacial aging are typically more advanced among Caucasian patients, exemplified in this figure by deeper rhytids and periocular hollowing.

Similarly, oculofacial aging patterns vary across racial demographics, some of which are illustrated in Figure 3. Caucasian patients generally have thinner skin and less subcutaneous fat compared to other racial groups, making them more vulnerable to the early onset of fine rhytids, particularly around the eyes and forehead [46]. These patients are also more affected by UV-induced damage due to lower melanin content, often presenting with dyspigmentation and textural changes [47]. Furthermore, Caucasian skin often has a thinner stratum corneum and reduced extensibility, which contributes to earlier signs of aging. Caucasian patients typically have more prominent brow ridges and deeper-set eyes, which may emphasize feature prominence with age-related facial volume loss [48]. Characteristic aging changes in Caucasians include fine rhytid formation in the periorbital and periocular regions, skin sagging, and jowl formation around the neck and chin [46,49].

In comparison, Asian patients have thick skin and more subcutaneous fat, which often delays the onset of fine lines and rhytids [46]. Research suggests that Asians have a weaker facial skeletal framework, which may make them more prone to midface soft tissue descent, malar fat pad ptosis, infraorbital hollowing, and tear trough formation [46]. Anthropometrically, South Asian individuals tend to have higher cheekbones and more prominent buccal fat [46]. Differences in facial skin characteristics also exist within Asian populations. For instance, Japanese individuals have shown increased skin moisture retention compared to other Asian groups [50]. There is also considerable variability in melanin content within Asian populations, with individuals from India generally having darker, more melanin-rich skin, while Japanese individuals tend to have the least melanin [46]. Further research is needed to be better understand subgroup differences within Asian populations.

Similarly, African individuals often display signs of aging at later ages compared to their Caucasian counterparts due to higher melanin content and a thicker stratum corneum [46,51]. They also have increased fibroblast activity, which helps maintain skin structural integrity [46,51]. In this group, aging may be associated with lesser midfacial bony volume loss than among Caucasian patients [52]. Additionally, concerns around hyperpigmentation and uneven skin tone tend to be more prominent [46].

Aging patterns among Hispanic and Latin American patients are less well characterized, though concerns related to skin mottling, infraorbital hollowness, shadowing, and jowl formation have been reported [53]. Similarly to individuals of Asian and African descent, they have increased melanin content, which reduces the effects of photoaging [37]. More research is needed to better understand the characteristic aging patterns in this population.

Research on facial aging patterns among Native American and Pacific Islander populations is limited, highlighting the need for further studies to better understand their unique signs of aging and to develop appropriate oculofacial interventions.

## 3. Perception of Oculofacial Aging

Especially among older adults, patients’ self-perceptions of aging are thought to influence physical, cognitive, and social functions [54]. Indeed, within this age group, the self-perception of aging is an important psychosocial factor that can affect perceived quality of life [55], with evidence supporting an association between a positive aging perspective and enhanced quality of life [55].

Evidence suggests that a growing range of patient demographics demonstrates an interest in oculofacial procedures. Between 2019 and 2022, facial surgical procedures increased by 18%, while the use of neuromodulating injections increased by 73% [56]. Although older patients often seek treatment to address advanced signs of aging, younger demographics are increasingly pursuing treatment to delay the aging process [57]. Indeed, one systematic review found that 21–43% of college-aged survey participants (more often young women) expressed interest in cosmetic procedures, with 1.3–6.4% reported previous cosmetic procedures [57]. Especially among young women, cosmetic procedure use is inversely associated with body satisfaction, and this relationship appears to be influenced by digital media perceptions, the value placed on physical appearance, the role of appearance in self-worth, and the influence of celebrities [57]. Of these, social media is an emerging factor; more time spent on such platforms and the development of negative self-perceptions are associated with an increased likelihood of undergoing elective cosmetic procedures [58]. As social media use rises, it is unsurprising that the prevalence of body dysmorphic disorder has also increased in the oculoplastic surgery setting, particularly among female and Caucasian patients [59]. However, while such patients are more likely to pursue cosmetic treatment, they are also more likely to express dissatisfaction with postoperative outcomes [60,61]. Clinicians should remain aware of the growing influence of cultural pressures in cosmetic procedures, especially among women who face greater societal pressures regarding body ideals promoted by media [62].

Evidence suggests that Caucasian women report lower levels of general body satisfaction compared to other racial groups, though demographic differences regarding the importance of facial features have been less thoroughly studied [63]. Some evidence indicates that Black or African American women report higher levels of body appreciation, potentially due to broader acceptance of beauty ideals that diverge from Eurocentric standards [63]. Although less studied, research suggests that Asian American women report similar levels of body dissatisfaction to Caucasian women and often internalize Eurocentric standards [63]. Beyond North American women, among Chinese women living in East Asia, one report suggests that Chinese subjects perceived Chinese faces to appear more youthful after the manipulation of dark facial spots than after wrinkling or skin sagging [64]. Comparatively, reports on body satisfaction among Hispanic or Latin American women are conflicting, with indications of either more favorable or comparable dissatisfaction levels to non-Hispanic women [63]. Although facial features are often highly valued by women, particularly among racialized groups, such features are rarely included in body image research [63].

## 4. Minimally Invasive Esthetic Interventions

Patient selection is paramount when considering facial rejuvenation procedures, whether incisional or non-incisional. This selection should be primarily informed by the collection of a comprehensive preoperative history and assessment. The patient’s chief concerns and expectations should be noted, along with the history of the presenting complaint (e.g., site, onset, associated symptoms, impact on function, and contributing factors to age-related changes). Relevant medical history (e.g., dry eye disease, anticoagulants, systemic conditions such as thyroid associated orbitopathy, previous facial traumas or procedures, and psychiatric history) should be identified. A comprehensive oculoplastic physical examination including the Fitzpatrick skin type should be performed. If indicated, further investigations may be needed to rule out pathological mimics of facial aging, especially for atypical-appearing cases.

Instead of surgical rhyridectomy, we will focus on the minimally invasive esthetic interventions used to treat oculofacial aging, including botulinum toxin injections (“Botox”), dermal fillers, energy-based treatments (e.g., lasers), and emerging techniques such as micro-focused ultrasound and platelet-rich plasma therapies.

### 4.1. Botulinum Toxin

Botulinum toxin was first identified in 1973 as an injectable substance capable of inducing local and prolonged muscle weakness [65]. Its mechanism involves the inhibition of acetylcholine release at the neuromuscular junction, with flaccid paralysis of the target muscle for months [65]. Early ophthalmic and oculofacial applications of botulinum toxin included treatments for strabismus (1979), blepharospasm (1982), and hemifacial spasm (1989) [66]. Since then, its use has expanded to treat glabellar rhytids, lateral canthal rhytids, upper nasalis rhytids, lower eyelid rhytids, eyebrow lifts, patients with thyroid-associated orbitopathy who have glabellar rhytids or lid retraction, use in adjunctive therapy to prolong CO_2_ laser treatment results [66], botulinum ptosis in patients with compromised corneas, and lacrimal gland injections for recalcitrant tearing. Botulinum toxin can also be used for perioral rhytids and platysma bands. In our experience, botulinum toxin has been useful for the treatment of dynamic rhytids at areas with crow’s feet, glabellar folds, and forehead wrinkling. Botulinum toxin is contraindicated in cases of active infection at injection sites, allergy, pregnancy or lactation, and in patients with a history of neuromuscular disorders (e.g., amyotrophic lateral sclerosis, myasthenia gravis, multiple sclerosis, and Eaton–Lambert syndrome) [67]. While botulinum toxin is not expected to cross the placental barrier, evidence of its safety during pregnancy is limited [67].

The effects of botulinum toxin injection typically manifest within one to four days post injection, and last three to six months, with maximum effect observed at one to four weeks [68]. As an oculofacial esthetic intervention, multiple studies report high patient satisfaction and improved psychological outcomes with botulinum toxin injections in short- and long-term contexts [69]. After five cycles of therapy, patients and providers report even higher satisfaction rates [67]. For patients with facial lines and disorders like strabismus, botulinum toxin therapy has also been shown to significantly improve quality of life [70,71].

For some patients, botulinum toxin may sufficiently reduce signs of oculofacial aging; however, it is a temporary treatment requiring repeated injections to maintain results. The interval between injections varies based on individual muscle anatomy, with most patients requiring reinjection every three to four months [68]. Some patients may also develop tolerance to botulinum toxin. In such cases, switching to another formulation or adjusting the dosage may be beneficial. Evidence suggests that up to 5–15% of patients using older botulinum toxin formulations repeatedly or at escalating doses developed neutralizing antibodies, reducing treatment effectiveness [72]. For this reason, the lowest effective dose with at least a one-month interval between injections is recommended [72].

The esthetic application of botulinum toxin injections carries a relatively favorable safety profile [73]. Reported localized side effects include pain, ptosis, lower eyelid drooping, ecchymosis, diplopia, muscle weakness, ectropion, hypesthesia, and headache [73,74,75]. Systemic adverse effects may include rashes, nausea, headache, fatigue, and flu-like symptoms [76].

### 4.2. Dermal Fillers

Dermal fillers are a minimally invasive option for reducing signs of periocular aging and can be classified based on their composition and durability [77]. They work by adding volume to and filling in soft oculofacial tissues, ultimately promoting facial rejuvenation and a more youthful appearance [78,79].

Esthetically, dermal fillers may be indicated for the tear trough area, upper eyelid, lateral canthal lines, glabellar rhytids, and brow [77]. In our experience, dermal fillers are often used for the treatment of nasolabial folding. These fillers are contraindicated in patients who are pregnant, breastfeeding, and hypersensitive or allergic to filler materials or lidocaine, or those with glabellar skin necrosis, a history of hypertrophic scarring, or keloid formation [78,80].

Several types of dermal fillers are commercially available, including hyaluronic acid (HA), autologous fat, polynucleotides (PNs), poly-L lactic acid (PLLA), calcium hydroxyapatite (CaHA), and polymethyl methacrylate (PMMA) [77,81]. Among these, HA is the most commonly used and studied, followed by calcium hydroxyapatite [81]. Numerous studies report both objective and subjective esthetic improvements with HA filler injections [82,83,84]. Generally, there are high levels of short- and long-term patient satisfaction following filler injection [77]. Specifically, periocular HA filler has been associated with patient satisfaction rates of 83.6% in the short term and 76.7% in the long term. One study evaluating CaHA fillers in 301 patients found significant improvements in appearance, while another study found that 93.5% of patients were satisfied with receiving CaHA fillers [85,86].

To maintain results, repeat filler injections may be necessary depending on patient factors and filler type. As temporary fillers, HA, CaHA, and PLLA last approximately 6–18 months, 12 months, and up to 2 years, respectively, though effects gradually diminish over time [81]. PMMA, a semi-permanent filler, may last up to 5 years, while autologous fat injections generally require fewer repeat injections [87].

Adverse events from filler are summarized in Table 2, and may range from mild and requiring minimal intervention to untreatable vision loss [77,88]. Adverse effects of dermal fillers include malar edema, granulomas, filler migration, xanthelasma, skin necrosis, vision loss (occasionally, at least 158 cases reported to date), anterior segment ischemia, and ophthalmoplegia, as shown in Table 1 [77,88]. Complications affecting vision can significantly impact overall quality of life [79]. Filler-induced vision loss may involve retrograde intravascular injection, followed by the anterograde blockage of an ocular perfusing artery, such as the central retinal artery [89]. Alternatively, severe ocular pain may occur several seconds after injection if the filler occludes the ophthalmic artery [89]. Most complications associated with HA not related to blindness or stroke can be effectively managed using the reversal agent hyaluronidase. There is no uniformly successful treatment for filler-induced vision loss but possible interventions include ocular massage, hyaluronidase, intravenous steroids, lowering the intraocular pressure, and hyperbaric oxygen therapy [90].

Glabellar injections should be performed with care to minimize the risk of skin necrosis and vision loss [91]. Hyaluronic acid injections are preferred. Carruthers et al. suggested that the needle should be placed superficially, parallel to the glabella, and injections should be performed in a retrograde fashion after aspiration to ensure no blood vessel entry [91]. There is some controversy on whether blunt cannula or sharp needles are preferred for the injection. Finally, ultrasonography can potentially aid in vascular mapping, the identification of previous filler, and to guide hyaluronidase injections for filler dissolution [92]. Given this, ultrasound can play a role in the prevention and assessment of procedural complications [92]. For instance, the technology can facilitate precise needle placement, permit real-time visualization of the injected area, and potentially detect early-onset of vascular complications [92]. For cases of vascular compromise, ultrasound may facilitate the safe dissolution of injected filler [92].

### 4.3. Energy-Based Treatments

Energy-based techniques, such as lasers (ablative and non-ablative) and intense pulsed light (IPL) therapies, can mitigate signs of periocular aging in a minimally invasive manner. Ablative laser therapies, including superpulsed or ultrapulsed carbon dioxide (CO_2_) and erbium: yttrium–aluminum–garnet (Er:YAG) lasers, are more commonly used than non-ablative lasers for periocular rejuvenation [93,94]. Non-ablative laser therapy is associated with reduced thermal damage and fewer side effects but has limitations in addressing deep oculofacial rhytids [95]. Ablative laser therapy involves removing the epidermis to eliminate aged skin and penetrating the dermis to stimulate collagen remodeling and heat-induced collagen contraction, promoting skin tightening [96]. Collectively, this leads to improved skin texture, tone, and a reduction in periocular rhytids [96]. Results can appear as early as four weeks post treatment and may last beyond one year [97].

Esthetic indications for oculofacial ablative laser therapy include periorbital wrinkles, facial resurfacing, dyschromia, and acne scars [98]. Laser skin resurfacing is a helpful complement to lower blepharoplasty and rhytidectomy. However, laser therapy is contraindicated in patients with continuous ultraviolet exposure, recent (<6 months) isotretinoin use, dermabrasion, collagen vascular disease, chemical peel procedures, and a history of hypertrophic scars, keloid formation, active or latent herpetic infections (latent being a relative contraindication and may warrant antiviral prophylaxis), or radiation therapy to the treatment site [98].

Laser therapy is generally associated with high patient satisfaction and quality of life improvements [99,100]. One study reported that 85.6% of patients receiving lower eyelid laser therapy were very satisfied with their esthetic outcomes [99]. Potential adverse events associated with ablative laser therapy include herpes simplex infection, thermal necrosis, ectropion and lagophthalmos, scarring, dyschromia, irritant dermatitis, and erythema [95,101]. These side effects can be mitigated using fractionated lasers that remove less than 60% of the epidermis via microablated columns with juxtaposed areas of untouched epithelium that facilitates faster recovery and re-epithelialization [94].

IPL therapy may also be used in the periocular region to address dyspigmentation, rhytids, skin laxity [102], blepharitis, and meibomian gland dysfunction. Unlike laser therapies, IPL uses pulses of light to downregulate inflammatory cytokines, reducing blood vessel inflammation in the periocular region [94]. Similarly to laser therapy, IPL induces collagen contraction, causing periocular skin to tighten [94]. IPL can improve esthetics, specifically in wrinkle appearance and hyperpigmentation [94]. A study evaluating IPL for periocular rejuvenation found that 51.5% of patients were moderately to considerably satisfied with facial rejuvenation outcomes [103]. Microneedling is a minimally invasive cosmetic procedure that involves the use of fine needles to create tiny, controlled punctures (micro-injuries) in the skin’s surface. This process is designed to stimulate the production of collagen and elastin and thereby improve the appearance of wrinkles, acne scars, and hyperpigmentation. Table 3 depicts a summary of energy-based treatments and microneedling techniques.

### 4.4. Emerging Techniques

Recently, new modalities for periocular aging have emerged, including micro-focused ultrasound (MFU) and platelet-rich plasma (PlatRP) therapies [104]. MFU enables skin tightening without damaging superficial skin by inducing small areas of thermal injury in targeted regions of the superficial dermis and subdermal connective tissue, which stimulates neocollagenesis and skin lifting [104,105,106]. MFU is indicated in patients with periocular rhytids, brow ptosis, mild to moderate skin laxity, and infraorbital hollowing [107,108]. Patients with moderate to severe skin laxity, however, may benefit more from surgical intervention [108]. MFU is contraindicated in patients with cystic acne, active infections, open wounds in the treatment area, and in pregnant patients [108]. A recent case series showed MFU to be relatively safe and well tolerated as a non-invasive approach, with no adverse effects reported among the patients [107]. However, other reports indicate that MFU therapy could result in pain, ecchymosis, transient paralysis, edema, striations, erythema, bruising, numbness, and dyspigmentation [108]. Some studies have found MFU to be effective within three to six months, though annual repeat sessions may be required to maintain optimal results [109,110,111]. Although MFU has demonstrated reasonable short-term safety and efficacy for rejuvenating periocular aging, further research is needed to assess its long-term efficacy and safety through high-quality studies that follow patients beyond one year [112]. Additionally, there is uncertainty regarding the optimization of treatment regimens. Specifically, high-quality research comparing MFU to other periocular esthetic treatments, or investigating its use in combination with them, remains limited [113].

PlatRP injections are another esthetic treatment that may help mitigate signs of periocular aging. PlatRP injections involve extracting plasma with a higher platelet concentration than that of normal circulating blood from whole blood [114]. PlatRP stimulates fibroblast proliferation in the dermis, matrix metalloproteinase expression, and collagen synthesis [105]. Moreover, the high concentration of platelets contains several growth factors involved in tissue repair, collectively enhancing skin quality and reducing signs of periocular aging [114]. PlatRP therapy may be used for mild to moderate periocular skin laxity, periocular hyperpigmentation, rhytids, and photoaging [115], and in our experience, it can be used as an adjunct to botulinum toxin, filler, or blepharoplasty. PlatRP therapy is absolutely contraindicated in patients with septicemia, critical thrombocytopenia, platelet dysfunction syndrome, and hemodynamic instability [116]. Side effects associated with PlatRP injections include edema, pain, erythema, bruising, and, rarely, blindness [116]. Before ultimately deciding on PlatRP therapy, the surgeon should gauge patients’ expectations and consider whether the postoperative result is likely to meet their satisfaction.

Studies evaluating PlatRP injections found that they (i) significantly improved periocular dark circles within 3 months of treatment, with effects lasting up to 6 months [117]; (ii) reduced infraorbital and lateral canthal rhytids without obvious side effects [118,119]; and (iii) enhanced skin barrier function, texture, elasticity, and smoothness [120,121]. PlatRP has also been shown to improve healing and has significant potential as an adjunctive therapy when used with other therapeutic modalities, such as fractional lasers [122,123]. While these studies demonstrate PLATRP’s effectiveness in periocular skin rejuvenation, further high-quality trials with longer follow-up periods are needed to optimize therapeutic regimens and evaluate the duration of esthetic improvements [124].

## 5. Challenges and Ethical Considerations

The minimally invasive esthetic interventions for periocular aging discussed above have shown significant potential; however, they present unique challenges and ethical considerations for both physicians and patients.

Firstly, assessing patient satisfaction in a standardized way is challenging in the field of esthetics due to the subjective nature of esthetic ideals and societal beauty standards. While validated patient-reported outcome measures, such as FACE-Q, FLO-11, and FLSQ, exist, they are often lengthy, potentially leading to patient fatigue and inaccurate self-reporting [125]. Furthermore, these assessments do not consistently capture the personalized needs of patients across diverse age ranges, races, ethnicities, and gender identities [126]. Individual variations in expectations, body image, and self-esteem can complicate objective assessment of patient satisfaction with esthetic procedures [127,128]. The periocular area is particularly sensitive, making it more susceptible to minor imperfections or complications like scarring, bruising, or swelling, which may occur during the recovery period [129]. As this area is a focal point in social interactions, patients may experience heightened levels of self-consciousness in response to these temporary issues [125,130]. This complicates the evaluation of long-term patient satisfaction, as satisfaction levels during recovery may differ from those post recovery [128]. Physicians may also struggle to assess overall patient satisfaction when patients focus on minor details rather than the broader effectiveness of esthetic intervention [128].

Managing patient expectations can be challenging, as esthetic outcomes are often subtle, whereas patients may expect substantial improvements [128]. Some patients may seek several treatments within a short timeframe to achieve their desired outcome; however, excessive treatment can result in unnatural appearances or irreversible structural issues, such as eyelid drooping, scarring, and erythema [94,131]. Therefore, it is essential for physicians to communicate realistic expectations, emphasizing that achieving complete symmetry or eliminating all signs of periocular aging may not be feasible [125]. Screening questionnaires to help identify patients with possible body dysmorphic disorder may be helpful. In such patients, prompt recognition and referral to a psychiatrist for counseling are crucial [132]. Educating patients about the potential risks and limitations of periocular esthetic procedure helps them set realistic expectations, helps them make informed decisions, and ultimately enhances satisfaction [125].

Social media can further exacerbate challenges related to periocular esthetic intervention. Social media often perpetuates body image concerns and unrealistic esthetic expectations due to images that may be filtered, digitally enhanced, or idealized [133]. Moreover, physicians who use social media to showcase successful cases may also raise ethical concerns. While promoting successful cases can benefit the practice, these images may not represent typical outcomes, potentially leading patients to pursue esthetic interventions based on unrealistic expectations [134]. To mitigate this effect, practitioners should limit the use of highly edited or unrepresentative images on social media [134].

## 6. Conclusions

Oculofacial aging, characterized by descent of the soft tissues and volume alterations, is a multifactorial process influenced by genetic, environmental, and lifestyle factors, and is characterized by the gradual onset of features such as rhytid formation, periorbital hollowing, and eyelid ptosis. This process progresses in distinct patterns across sex- and race-based patient subgroups. For instance, compared to Black patients, Caucasians tend to exhibit earlier signs of periocular aging due to thinner dermal layers and lower melanin content. Despite these insights, there remains a paucity of representative research among underrepresented populations, including Hispanic, Native American, and Pacific Islander groups.

Currently available, minimally invasive esthetic interventions for periocular aging include botulinum injections, dermal fillers, and energy-based therapies, which have demonstrated considerable safety and efficacy for oculofacial rejuvenation in both the short and long term. More recently, MFU and PlatRP therapies have also shown promise for short-term safety and efficacy; however, high-quality research on their long-term effects and optimal administration regimens is limited. Patient selection is paramount when considering facial rejuvenation procedures, whether incisional or non-incisional. Further high-quality randomized controlled trials are needed to clarify the benefits, risks, and limitations of these novel therapies in comparison to conventional treatments, ultimately optimizing esthetic outcomes for patients.

## Figures and Tables

**Table 1:** Summary of skin-related changes from common pathophysiological contributors to periorbital aging.

Contributor	Class	Mechanism
Sun Exposure	Modifiable	DNA damage, carcinogenesis, and degradation of the extracellular matrix
Extreme Climate	Modifiable	Trans-epidermal water loss and moisture depletion
Air Pollution	Modifiable	Generation of reactive oxygen species
Smoking	Modifiable	May be related to cutaneous vasoconstriction, impaired extracellular matrix quality
Diet	Modifiable	Retention of moisture, supplementation of antioxidants and carotenoids
Chronic Stress and Poor Sleep	Modifiable	Promotion of inflammation- and oxidation-related skin changes
Repetitive Facial Expressions	Modifiable	Repetitive reinforcement of dynamic wrinkling
Extracellular Matrix	Genetic	Anomalous or deficient matrix proteins may reduce resistance to aging
Innate Resistance to Inflammatory and Oxidative Stressors	Genetic	Impaired resistance to inflammation- or oxidation-related skin damage
Cutaneous Melanin	Genetic	Protective against ultraviolet radiation
Cutaneous Melanin	Genetic	Protective against ultraviolet radiation

**Table 2:** Notable adverse events of dermal fillers.

Adverse Effect	Incidence Rate	Associated Dermal Filler	Management
Malar Edema	11% (periocular HA)	Hyaluronic acid (HA)	Hyaluronidase, multiple treatments may be needed
Granuloma	0.02–1% (facial injections)	HA, PMMA, PLLA	Intralesional steroids, hyaluronidase (for HA), filler removal, surgical excision (for PMMA, PLLA)
Migration	0.02–1%	HA	Hyaluronidase
Xanthelasma	Not specified	Various dermal fillers	No successful treatment identified
Skin Necrosis	0.00001%	HA	Antibiotics, oral steroids, hyaluronidase, debridgement
Vision Loss	At least 158 reported cases in 2024	HA, AF, CaHA, PLLA	Hyaluronidase (for HA), varied delivery methods

Abbreviations: polymethyl methacrylate (PMMA); poly-L lactic acid (PLLA), autologous fat (AF); calcium hydroxyapatite (CaHa).

**Table 3:** Summary of energy-based treatments and microneedling techniques.

Technique	Indications	Advantages	Limitations	Complications
Ablative Lasers	Periorbital wrinkles, facial resurfacing, dyschromia, acne scars, adjunct to blepharoplasty	Results often persist over one year, improved skin texture and tone, and effective for deep rhytids	Contraindications include ultraviolet exposure, recent isotretinoin use, dermabrasion, collagen vascular disease, chemical peel, keloid scars, herpetic infections, and radiation therapy	Herpes simplex reactivation, thermal necrosis, ectropion, lagophthalmos, scarring, dyschromia, irritant dermatitis, and erythema. Fractional lasers reduce these risks.
Non-ablative Lasers	Periorbital rejuvenation	Reduced the risk complications (e.g., thermal damage) compared to ablative lasers	Limited in treatment of deep oculofacial rhytids	Complications are generally milder compared to ablative lasers, and may include mild redness or irritation.
Intense Pulsed Light (IPL)	Dyspigmentation, rhytids, skin laxity, blepharitis, meibomian gland dysfunction	Non-invasive and effective for improving wrinkles, hyperpigmentation, and skin texture	Patient satisfaction rates may be lower than with laser therapy, and it is less effective for deep wrinkles or extensive skin laxity	Side effects are generally minimal, including mild irritation or discomfort during treatment.
Microneedling	Wrinkles, acne scar, hyperpigmentation	Minimally invasive procedure with the ability to improve skin tone and texture over multiple sessions	May not be suitable for individuals with active infections, inflammatory skin conditions, or poor wound healing capacity	Common complications include minor erythema and irritation at the treatment site

## Data Availability

Not data were created in the production of this work.
